# Integrated analysis of gene expression and DNA methylation changes induced by hepatocyte growth factor in human hepatocytes

**DOI:** 10.3892/mmr.2015.3974

**Published:** 2015-06-22

**Authors:** CHENG-RONG XIE, HONGGUANG SUN, FU-QIANG WANG, ZHAO LI, YI-RUI YIN, QIN-LIANG FANG, YU SUN, WEN-XIU ZHAO, SHENG ZHANG, WEN-XING ZHAO, XIAO-MIN WANG, ZHEN-YU YIN

**Affiliations:** Department of Hepatobiliary Surgery, Zhongshan Hospital, Xiamen University, Fujian Provincial Key Laboratory of Chronic Liver Disease and Hepatocellular Carcinoma, Xiamen, Fujian 361004, P.R. China

**Keywords:** hepatocellular carcinoma, hepatocyte growth factor, DNA methylation, tumor suppressor gene

## Abstract

Hepatocellular carcinoma (HCC) is the one of most common malignant tumors. The tumor microenvironment has a role in not only supporting growth and survival of tumor cells, but also triggering tumor recurrence and metastasis. Hepatocyte growth factor (HGF), one of the important growth factors in the tumor microenvironment, has an important role in angiogenesis, tumorigenesis and regeneration. However, the exact mechanism by which HGF regulates HCC initiation and development via epigenetic reprogramming has remained elusive. The present study focused on the epigenetic modification and target tumor-suppressive genes of HGF treatment in HCC. Expression profiling and DNA methylation array were performed to investigate the function of HGF and examine global genomic DNA methylation changes, respectively. Integrated analysis of gene expression and DNA methylation revealed potential tumor suppressor genes (TSGs) in HCC. The present study showed the multiple functions of HGF in tumorous and nontumorous pathways and global genomic DNA methylation changes. HGF treatment upregulated the expression of DNA methyltransferase 1 (DNMT1). Overexpression of DNMT1 in HCC patients correlated with the malignant potential and poor prognosis of HCC. Furthermore, integration analysis of gene expression and DNA methylation changes revealed novel potential tumor suppressor genes TSGs including *MYOCD*, *PANX2* and *LHX9*. The present study has provided mechanistic insight into epigenetic repression of TSGs through HGF-induced DNA hypermethylation.

## Introduction

Hepatocyte growth factor/scatter factor (HGF/SF), which is mainly secreted by Kuppfer cells, endothelial cells, fibroblasts and hepatic stellate cells in the liver, has been shown to have a role in embryonic organ development, adult organ regeneration and wound healing (1). HGF regulates cell growth, cell motility and morphogenesis by binding to its unique receptor c-Met and by activating a tyrosine kinase-signaling cascade (2–4). Owing to its functions, including stimulating mitogenesis, cell motility and matrix invasion, HGF has a central role in angiogenesis, tumorigenesis and tissue regeneration.

Hepatocellular carcinoma (HCC) is one of most common malignant tumors; the incidence rate and mortality of HCC is the fifth- and third-highest in the world, respectively (5). Although several advanced treatments are now available, including surgical resection, liver transplantation and ablation therapies (6), and in spite of the development of molecular-target drugs such as sorafenib (7), the five-year overall survival rate of patients with advanced HCC remains poor (8). The main reason for the poor survival rate of advanced HCC is the high rate of recurrence and metastasis after local treatment (9).

The tumor microenvironment is composed of various stromal cells, including myofibroblasts, vascular cells and immune cells, and it has an important role in not only supporting the growth and survival of tumor cells but also in triggering tumor recurrence and metastasis (10). Within the tumor microenvironment, several of the growth factors secreted by stromal or tumor cells, including HGF, insulin-like growth factor 1 (IGF1), epidermal growth factor (EGF), vascular endothelial growth factor (VEGF) and platelet-derived growth factor (PDGF), may induce a similar signaling cascade downstream of receptor tyrosine kinase (RTK) and trigger synergistic tumor recurrence and metastasis (11). The important role of the HGF/c-Met signaling pathway in carcinogenesis and development has been well established. For example, this pathway activates mitogen-activated protein kinase (MAPK) and phosphoinositide 3-kinase (PI3K)/AKT/mammalian target of the rapamycin (mTOR) pathways as well as enables cross-talk with epidermal growth factor and transforming growth factor (TGF)-β ligands. Overactivation of these pathways promotes proliferation, survival, migration, invasiveness and angiogenesis of certain tumors (11,12). In general, the HGF expression levels in the liver cancer microenvironment after surgery show marked increases, implicating HGF as one of the main causes of HCC recurrence and metastasis (13).

In addition to genetic alteration, abnormality of epigenetic mechanisms is also frequent in carcinogenesis and cancer progression, including dysregulation of histone modification and DNA methylation (14). DNA methylation is one of the most important epigenetic mechanisms that modulate gene expression in a plethora of physiological and pathological processes, including carcinogenesis (15). Accurate DNA methylation patterns are established by the *de novo* DNA methyltransferases (DNMTs) DNMT3A and DNMT3B, and then subsequently maintained by DNMT1 (16). DNMTs are overexpressed in various cancer types, including colorectal (17), prostate (18), hepatocellular (19), breast (20), gastric (21) and lung cancers (22), and their overexpression is significantly correlated with poor histological differentiation and prognosis (17–22). At present, it is generally accepted that tumor environmental cues together with cell-intrinsic alterations contribute to the epigenetic changes in carcinogenesis as well as recurrence and metastasis of cancer, as these changes induce adaptations of cancer cells for successful invasion of the stroma, entry and survival in the lymphatic or blood vessels, followed by spread and colonization of distant or different organs (23,24). It is thus clear that the tumor micro-environment, particularly the growth factors, are able to affect the functions of epigenetic molecules. However, the potential tumor suppressor genes (TSGs) regulated by HGF via an epigenetic mechanism have remained to be identified. The present study investigated the target TSGs of HGF by altering DNA methylation.

## Materials and methods

### Ethics statement

The present study was approved by the Xiamen University Medical Ethics Committee (Xiamen, China), and written informed consent was obtained from all participants or their representatives if direct consent could not be obtained.

### Cell culture and reagents

The HCC cell line HepG2 was obtained from the Type Culture Collection of the Chinese Academy of Sciences (Shanghai, China) and cultured in Dulbecco's modified Eagle's medium (DMEM; Gibco-BRL, Invitrogen Life Technologies, Carlsbad, CA, USA). The immortalized normal human liver cell line HL-7702 was obtained from the China Center for Type Culture Collection (Wuhan, China) and cultured in RPMI-1640 medium (Gibco-BRL). Media were supplemented with 10% fetal bovine serum (FBS; Hyclone, Logan, UT, USA) and 1% penicillin-streptomycin (Gibco-BRL). The cells were incubated at 37°C in an incubator with 5% CO_2_. Recombinant human HGF was obtained from Millipore (Billerica, MA, USA). 5′Aza-2′-deoxycytidine (5′Aza) was obtained from Sigma-Aldrich (St Louis, MO, USA).

### Tissue samples and clinical characteristics of patients

Tumor samples were obtained from the Chronic Liver Disease Biological Tissue Bank, Department of Hepatobiliary Surgery, Zhongshan Hospital of Xiamen University (Xiamen, China), which were obtained during surgery between 2008 and 2011. Paraffin blocks of tumor tissue from 89 patients were prepared for immunohistochemical (IHC) assays. The patients were aged between 30 and 70 and 72 were male and 17 were female. The patients were assessed at two-month intervals after surgery in outpatient clinics or by routine telephonic inquiry. The end of the follow-up period was December 2011 for all patients. The overall survival was calculated from the day of surgery to the day of succumbing to the disease or final follow-up.

### RNA isolation and reverse transcription quantitative polymerase chain reaction (RT-qPCR)

Total RNA was extracted with the TRIzol reagent (Invitrogen Life Technologies). cDNA was synthesized using the PrimeScript RT Reagent kit with gDNA Eraser (Takara Bio, Inc., Otsu, Japan). qPCR was performed using the StepOne™ Real-Time PCR system (Applied Biosystems, Foster City, CA, USA), SYBR^®^ Green (Takara Biotechnology Co., Ltd., Dalian, China) and SYBR Select Master mix (Takara Bio, Inc., Otsu, Japan) with the following cycling condiditons: 95º°C for 30 sec and 60°C for 1 min. The primers were designed and synthesized by BGI company (Shenzhen, China). β-actin mRNA was used as an endogenous control. The relative expression of RNA was calculated using the comparative Ct method (2^−ΔΔCT^).

### Microarray analysis

Expression profiling analysis was performed on HL-7702 cells and HGF-treated HL-7702 cells. To simulate hepatocarcinogenesis and to explore the long-term effect of HGF, HL-7702 cells were cultured in RPMI-1640 medium supplemented with 1% FBS and 50 ng/ml HGF for 4 weeks. The medium was replaced every day with fresh medium containing the same concentration of HGF. Total RNA from each sample was quantified by using the NanoDrop ND-1000 spectrometer (Thermo Fisher Scientific), and the RNA integrity was assessed by standard denaturing agarose gel electrophoresis (Biowest SAS, Nuaillé, France). The Human GeneArray 1.0 ST platform (Agilent Technologies, Santa Clara, CA, USA) was used for the microarray analysis. The sample preparation and microarray hybridization were performed based on the manufacturer's instructions. The labeled cRNAs were hybridized onto the Whole Human Genome Oligo Microarray (4×44K; Agilent Technologies). The slides were washed and the arrays were scanned by the Agilent Scanner G2505B (Agilent Technologies). Agilent Feature Extraction software (version 10.7.3.1; Agilent Technologies) was used to analyze the acquired array images. Raw signal intensities were normalized using the quantile method in the GeneSpring GX software (version 11.5.1; Agilent Technologies), and low-intensity genes were filtered. P<0.05 was considered to indicate significant changes in gene expression and when >2.0-fold changes in the ratio of means were observed.

### Methylation arrays

Genome DNA was extracted and bisulfite-converted using the Epitect Fast DNA Bisulfite kit (Qiagen, Hilden, Germany). DNA methylation microarray analysis was performed at Kangchen Biotech Inc. (Shanghai, China).

### Statistical analysis

All statistical analyses were performed using the SPSS software (version 19.0) for Windows (International Business Machines, Armonk, NY, USA). The survival curves were calculated by the Kaplan-Meier method, and comparison was performed by a log rank test. Values for parametric variables are expressed as the mean ± standard error. P<0.05 was considered to indicate a statistically significant difference between values.

## Results

### Gene expression profiling reveals multiple functions of HGF

**Figure 1:**
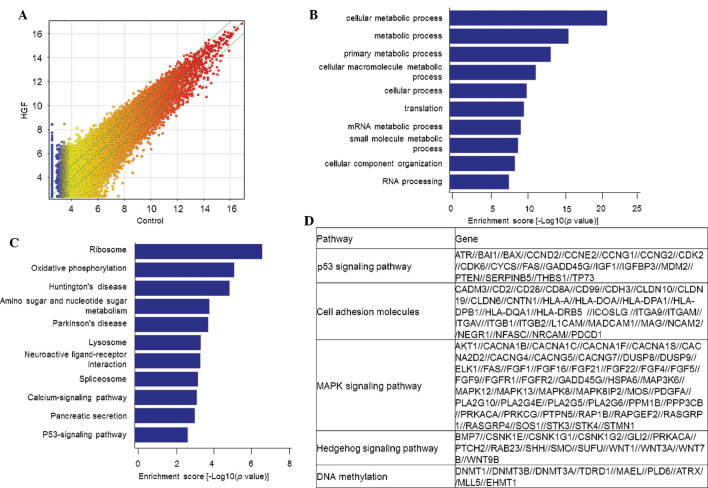
HGF treatment in HL-7702 cells induced a dramatic change in the gene expression profile. (A) Scatterplot for assessing the variation between two groups. Upper and lower oblique lines mean fold changes of >2 or <2, respectively. (B and C) A total of 6,874 genes (>2-fold change; P<0.05) were subjected to (B) gene ontology analysis and (C) pathway analysis. (D) The genes were classified into functionally associated gene groups by using the functional annotation clustering tool. HGF, hepatocyte growth factor.

To explore the underlying molecular mechanisms of HGF-induced carcinogenesis, the transcriptome following HGF treatment was examined using a microarray analysis. Comparison of the transcriptomes of untreated HL-7702 and HGF-treated HL-7702 cells identified 6,874 genes with a >2-fold change (P<0.05), of which 2,902 genes were upregulated and 3,972 genes were downregulated (Fig. 1A).

To obtain further insight into the biological function of the differentially expressed genes, 6,874 genes were subjected to Gene Ontology (GO) analysis by using the GeneSpring GX software. Unexpectedly, the genes were markedly enriched in multiple metabolic processes, including cellular, macromolecular and mRNA metabolic processes, as well as translation (Fig. 1B). The genes associated with metabolism were found to be significantly affected by HGF. Next, a pathway analysis was performed based on differentially expressed genes by using the latest Kyoto Encyclopedia of Genes and Genomes database (http://www.genome.jp/kegg/). Consistent with those of the GO analysis, the results of the pathway analysis revealed that metabolic pathways involving ribosomes, oxidative phosphorylation, amino sugars and nucleotide sugar metabolism had high enrichment scores, with a significance in the order in which they are stated (Fig. 1C). The genes were also markedly enriched in the P53 pathway, which is closely associated with HCC, suggesting that HGF influences the recurrence and development of HCC, mainly through the P53 pathway (Fig. 1C). A list of cancer-associated pathways showing significantly up- or downregulated genes is shown in Fig. 1D, including P53, cell adhesion molecules as well as the Hedgehog- and MAPK signaling pathways. However, HGF was also shown to influence several non-tumorous pathways, including those involved in Parkinson's and Huntington's disease, implying that HGF may have a role in nervous system diseases (Fig. 1C). The findings of the present study suggest that HGF may have multiple roles in these signaling pathways in HCC or in diseases of the nervous system.

### HGF induces DNMT1 overexpression in HCC patients, which correlates with the malignant potential and poor prognosis of HCC

**Figure 2:**
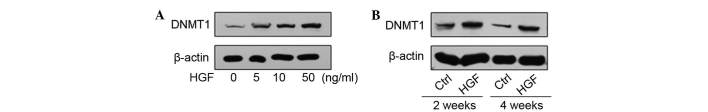
HGF upregulates DNMT1 protein levels. (A and B) Expression levels of DNMT1 in HL-7702 cells treated with HGF were analyzed by western blotting as a function of (A) dose and (B) time. HGF, hepatocyte growth factor; DNMT, *de novo* DNA methyltransferase; Ctrl, control.

**Figure 3:**
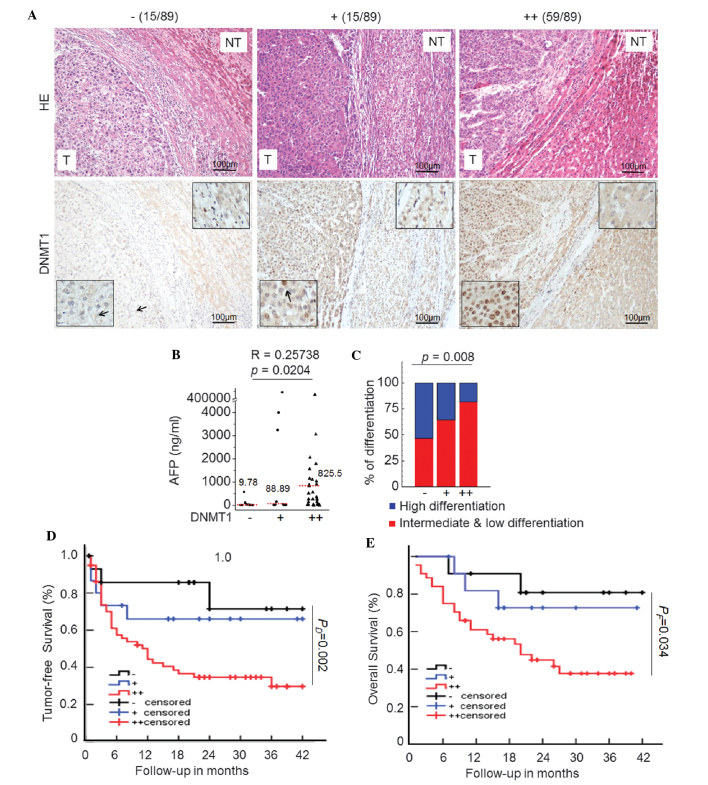
Upregulated DNMT1 expression correlates with malignant indicators of HCC. (A) Immunohistochemical staining of DNMT1 (brown) in paraffin sections of cancer tissues and adjacent tissues from HCC patients (scale bar, 100 *µ*m). According to the DNMT1 protein expression levels, 89 cases were divided into three groups (negative, moderately positive and strongly positive). (B) Scatter plots of serum AFP levels in the three study groups. In each panel, the dotted line indicates the median (r=0.26, P=0.02; Spearman correlation). (C) Distribution of histopathological differentiation and classification in the three study groups. The differentiation status is indicated as follows: Blue, high differentiation; red, intermediate and low differentiation. Differences in differentiation among the groups were significant (P=0.008). The Kaplan-Meier curves for (D) tumor-free survival and (E) overall post-operative survival indicate that HCC patients with marked DNMT1 overexpression had a worse prognosis as compared with those in the negative group. Arrows indicate representative cells. DNMT, *de novo* DNA methyltransferase; HCC, hepatocellular carcinoma; AFP, alpha-fetoprotein; H&E, hematoxylin and eosin; T, tumorous; NT, non-tumorous.

The results revealed that several differentially expressed genes induced by HGF treatment had a role in the DNA methylation pathway (Fig. 1D). DNMT1 is closely associated with various cancers; it is a key regulator in DNA hypermethylation (17). The DNMT1 protein levels was obviously increased following HGF treatment in the present study (Fig. 2). To unravel the clinical significance of abnormal DNMT1 expression in HCC, 89 primary HCC samples were examined by IHC staining. The results revealed that DNMT1 was more easily detectable in the nuclei of tumorous tissues than in paired non-tumorous tissues in 74 out of 89 (83.1%) HCC cases. Among these, 59 (66.2%) cases were strongly positive (++) and 15 (16.8%) were moderately positive (+) (Fig. 3A). Furthermore, a correlation analysis between DNMT1 expression and clinical features was performed for the 89 HCC cases. Elevated levels of serum alpha-fetoprotein (AFP) indicate a high risk of liver cancer (21). As shown in Fig. 3B, the serum AFP levels were significantly elevated in the DNMT1-overexpressing groups, with median serum AFP levels of 9.87, 88.89 and 825.5 ng/ml in the DNMT1-negative, -moderately positive and -strongly positive groups, respectively (P=0.02). The proportion of low and intermediate differentiation gradually increased with DNMT1 overexpression (Fig. 3C; P=0.008). Furthermore, Kaplan-Meier analysis revealed that the three-year recurrence-free survival rate was significantly lower for HCC patients in the strongly positive DNMT1 group as compared with that in the DNMT1-negative HCC patients (Fig. 3D; P=0.002). The three-year overall survival rate of patients with DNMT1-positive HCC was also significantly lower than that of patients with DNMT1-negative HCC (Fig. 3D; P=0.034). These cumulative findings suggested the clinical significance of DNMT1 as a biomarker for HCC diagnosis as well as prognosis.

### HGF induces changes in DNA methylation

**Figure 4:**
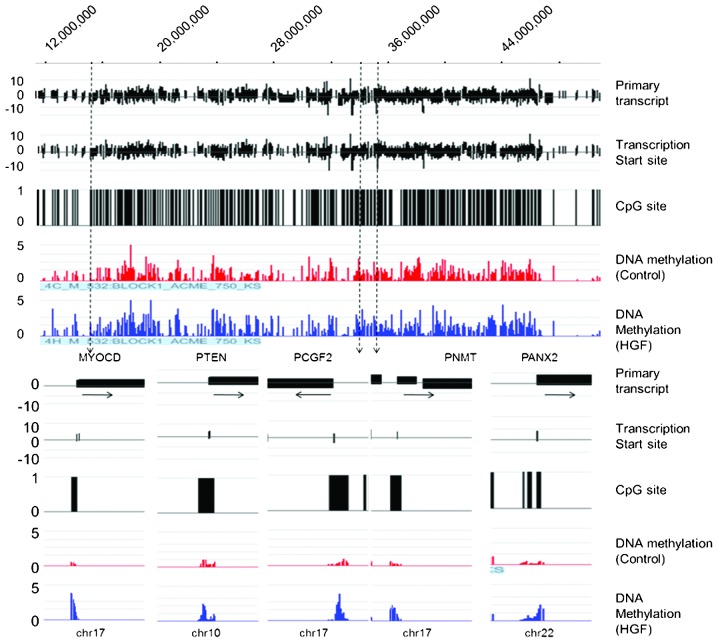
Genome-wide analysis of CpG island methylation of HL-7702 with or without HGF treatment. DNA methylation pattern on chromosome 17 (top) and the expanded view of selected positive signals for tumor suppressors from chromosome 17 and other chromosomes (bottom). HGF, hepatocyte growth factor; chr, chromosome.

Extensive low-level methylation of genomic DNA is a characteristic of a malignant tumors (25). Silencing of TSGs due to promoter hypermethylation is closely associated with tumorigenesis (26). To explore the effect of HGF on genomic methylation, a genome-wide DNA microarray analysis was performed. The level of DNA methylation in a number of loci was significantly altered in the HGF group as compared to that in the control group (Fig. 4). After HGF treatment, a total of 2,878 genes were hypermethylated and 2,302 genes were hypomethylated. Parts of known TSG promoters, including *PCGF2*, *PTEN*, and *PANX2*, were markedly hypermethylated following HGF treatment (Fig. 4). These results suggested that HGF may alter the expression of TSGs via an epigenetic mechanism involving DNA hypermethylation.

### Integrated analysis of gene expression and DNA methylation reveals potential TSGs in HCC

**Figure 5:**
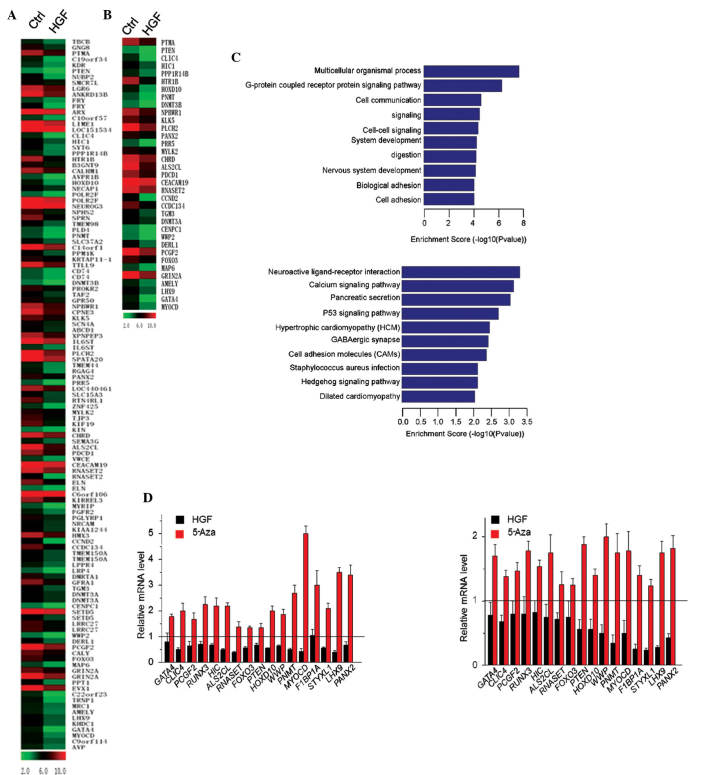
Integrated analysis of gene expression and DNA methylation. (A and B) Expression heatmap of genes regulated by HGF and DNA methylation in HL-7702 cells. (A) A total of 89 genes were screened out; (B) 35 of these were associated with cell proliferation, apoptosis, cell cycle, and metastasis. (C) The 89 potential target genes that were subjected to gene ontology analysis (top) and pathway analysis (bottom). (D) Quantitative real-time polymerase chain reaction assays were performed following 5′Aza and HGF treatment of HL-7702 (left) and HepG2 (right) cells. The relative levels of mRNA between treated cells vs. untreated cells were normalized to beta-actin. Values are expressed as the mean ± standard error. HGF, hepatocyte growth factor; 5′Aza, 5′Aza-2′-deoxycytidine.

One of the most important contributors to tumorigenesis is the downregulation of TSGs by hypermethylation of CpG islands (27). To reveal the epigenetic mechanism of HGF-mediated repression of TSG transcription via DNA hypermethylation, DNA methylation and gene expression profiles were subjected to integrated analysis. The results showed that the expression of a total of 89 genes was decreased by promoter hypermethylation after HGF treatment (Fig. 5A). 35 of these differentially expressed genes, were associated with cell proliferation, apoptosis, cell cycle and metastasis based on previous studies (Fig. 5B) (28–30). In addition, GO analysis revealed that the 89 differentially expressed genes were markedly enriched in the adhesion process (Fig. 5C), which is consistent with the findings reported by a previous study asserting that HGF promotes tumor metastasis (31). Furthermore, pathway analysis showed that the P53 pathway, hedgehog pathway and cell adhesion (Fig. 5C), which influenced HCC occurrence and metastasis, also had high enrichment scores. The 35 potential TSGs, which were associated with cell proliferation, apoptosis, cell cycle, and metastasis, were selected for the confirmation of microarray profiles in the HL-7702 and HepG2 cell lines. Among these 35 genes, the expression of 17 genes was downregulated following HGF treatment for 4 days, whereas these genes were upregulated following treatment with the DNA demethylation agent 5′Aza (Fig. 5D). The expression levels of *PTEN*, *PNMT*, *MYOCD*, *LHX9* and *PANX2* were obviously changed after HGF or 5′Aza treatment. These results identified potential TSGs regulated by HGF via DNA hypermethylation in HCC.

## Discussion

Metabolism is a fundamental aspect of every essential cell function. Metabolic reprogramming induced by growth factors including vascular endothelial growth factor-B has also been reported recently (32). The initiation and development of cancer is frequently associated with the upregulation of catabolic pathways (33). One such important pathway is aerobic glycolysis that preferentially metabolizes glucose to lactate, even in the presence of excess oxygen, and provides substances which are essential for cancer cell survival (33). In the present study, gene expression profiling demonstrated that HGF mainly participates in cellular metabolic processes, including macromolecular, mRNA- and translation-associated metabolic processes. HGF is a growth factor that exerts multiple effects; it promotes cell proliferation, migration and angiogenesis in cancer (34). Previous studies have mainly focused on dysregulation of certain tumor-associated signaling pathways influenced by HGF (35). Therefore, little is known about the effects of HGF-induced metabolic reprogramming on cancer development and recurrence. The present study provided novel clues for the exploration of the underlying mechanisms of HGF-induced tumorigenesis from a metabolic perspective.

Emerging evidence suggests an association between DNA hypermethylation and hepatocarcinogenesis (36). For example, DNMT1 overexpression was observed in 43% of HCC cases whose three-year overall survival rate was <40% (19). However, the mechanistic and prognostic significance of DNA hypermethylation in human HCC has remained to be elucidated. The findings of the present study demonstrated the clinical significance of aberrant DNA methylation in HCC diagnosis and prognosis. In 82.8% of HCC samples, DNMT1 protein was overexpressed. The results also revealed a significant positive association between DNMT1 overexpression and poor HCC prognosis. The Kaplan-Meier curves for post-operative overall survival indicated a worse prognosis for HCC patients with DNMT1 overexpression compared with those negative for DNMT1. The three-year overall survival rate was only 40% in the DNMT1-positive group, which was significantly lower (P=0.002) than that in the DNMT1-negative group (~80%). Serum AFP is currently the most widely used sero-logical tumor marker for HCC diagnosis. In the present study, median AFP levels were 9.78, 88.89 and 825.5 ng/ml in the DNMT1-negative, -moderately positive and -strongly positive group, respectively, and these differences were significant (P=0.0204). The normal range for AFP is 10–20 ng/ml, and a level >400 ng/ml is usually considered diagnostic for HCC. In the present study, the percentage of patients with AFP levels >400 ng/ml was 54.5% in the strongly positive group, which was significantly higher than that in the negative group (16.7%), suggesting that DNMT1 is clinically significant as an additional indicator in routine HCC diagnosis.

Evidence of epigenetic dysregulation in cancer, including aberrant histone modification and DNA methylation, has been rapidly expanding (37). The epigenetic reprogramming by the tumor microenvironment has a critical role in tumor growth and survival. Growth factors bind to their corresponding receptors and subsequently activate downstream signaling cascades. More importantly, these signaling pathway activations give rise to comprehensive epigenetic reprogramming. For example, TGF-β is an important factor that regulates proliferation, apoptosis, extracellular matrix composition and epithelial mesenchymal transition (38) through upregulation of SNAIL and TWIST family proteins and through recruitment of epigenetic molecules, including G9a, DNMTs (39), BMI1 (40) and EZH2 (41). However, the exact underlying mechanisms of HGF on tumor initiation and development through epigenetic reprogramming remain elusive. The present study demonstrated that HGF can alter the global chromosomal DNA methylation status. The methylation of promoters of certain known TSGs, including *PCGF2*, *PTEN* and *PANX2*, were enriched after HGF treatment. Methylation of the cytosine residues in the DNA of TSGs has been recognized as a silencing mechanism of fundamental importance in tumorigenesis and metastasis (42). DNA methylation and gene expression profiles were subjected to integration analysis to finally identify the following potential TSGs silenced by HGF via DNA methylation changes: *PTEN*, *PNMT*, *MYOCD*, *LHX9* and *PANX2*. *MYOCD* encodes a nuclear protein functioning as a transcriptional co-activator of serum response factor (SRF) and modulates the expression of cardiac and smooth muscle-specific SRF-target genes (43). However, to the best of our knowledge, there is no evidence of any role of MYOCD in any type of cancer; this aspect therefore requires further study. *LHX9*, which encodes LIM-homeodomain 9 transcription factor, was found to be involved in cell differentiation of several neural cell types (44). It has been reported that *LHX9* expression was reduced by promoter hypermethylation in malignant childhood gliomas. Restoration of *LHX9* expression inhibited glioma cell migration and invasion, suggesting the implication of LHX9 on the migratory phenotype of cancer (45). Panx2, which is a member of the gap-junction protein family, showed an overall reduction in gliomas and can thus help predict post-diagnosis survival of patients with glial tumors. In addition, restoration of Panx2 reduces oncogenicity *in vivo* and *in vitro* (46). *MYOCD*, *LHX9* and *PANX2* may thus be novel TSGs that are regulated by the HGF-DNA methylation pathway in HCC.

In conclusion, the results of the present study suggested a significant disruption of TSGs by hypermethylation. Several of these genes have not been previously implicated in HCC. To the best of our knowledge, the present study was the first to associate aberrant DNA methylation with gene expression induced by HGF in order to identify potential TSGs in HCC. The findings of the present study are valuable in the identification of key TSGs.

## References

[1] Nakamura T (1991). Structure and function of hepatocyte growth factor. Prog Growth Factor Res.

[2] Matsumoto K, Nakamura T (1992). Hepatocyte growth factor: Molecular structure, roles in liver regeneration and other biological functions. Crit Rev Oncog.

[3] Pavan S, Musiani D, Torchiaro E, Migliardi G, Gai M, Di Cunto F, Erriquez J, Olivero M, Di Renzo MF (2014). HSP27 is required for invasion and metastasis triggered by hepatocyte growth factor. Int J Cancer.

[4] Weidner KM, Hartmann G, Sachs M, Birchmeier W (1993). Properties and functions of scatter factor/hepatocyte growth factor and its receptor c-Met. Am J Respir Cell Mol Biol.

[5] Caldwell S, Park SH (2009). The epidemiology of hepatocellular cancer: from the perspectives of public health problem to tumor biology. J Gastroenterol.

[6] Belghiti J, Fuks D (2012). Liver resection and transplantation in hepatocellular carcinoma. Liver Cancer.

[7] Palmer DH (2008). Sorafenib in advanced hepatocellular carcinoma. N Engl J Med.

[8] Kishi Y, Hasegawa K, Sugawara Y, Kokudo N (2011). Hepatocellular carcinoma: Current management and future development-improved outcomes with surgical resection. Int J Hepatol.

[9] Ueno M, Uchiyama K, Ozawa S, Hayami S, Shigekawa Y, Tani M, Yamaue H (2011). Adjuvant chemolipiodolization reduces early recurrence derived from intrahepatic metastasis of hepatocellular carcinoma after hepatectomy. Ann Surg Oncol.

[10] Park CC, Bissell MJ, Barcellos-Hoff MH (2000). The influence of the microenvironment on the malignant phenotype. Mol Med Today.

[11] Hu CT, Wu JR, Wu WS (2013). The role of endosomal signaling triggered by metastatic growth factors in tumor progression. Cell Signal.

[12] Scagliotti GV, Novello S, von Pawel J (2013). The emerging role of MET/HGF inhibitors in oncology. Cancer Treat Rev.

[13] Osada S, Kanematsu M, Imai H, Goshima S (2008). Clinical significance of serum HGF and c-Met expression in tumor tissue for evaluation of properties and treatment of hepatocellular carcinoma. Hepatogastroenterology.

[14] Reik W (2007). Stability and flexibility of epigenetic gene regulation in mammalian development. Nature.

[15] Robertson KD (2005). DNA methylation and human disease. Nat Rev Genet.

[16] Johnson AA, Akman K, Calimport SR, Wuttke D, Stolzing A, de Magalhães JP (2012). The role of DNA methylation in aging, rejuvenation and age-related disease. Rejuvenation Res.

[17] Ting AH, Jair KW, Suzuki H, Yen RW, Baylin SB, Schuebel KE (2004). CpG island hypermethylation is maintained in human colorectal cancer cells after RNAi-mediated depletion of DNMT1. Nat Genet.

[18] Patra SK, Patra A, Zhao H, Dahiya R (2002). DNA methyltransferase and demethylase in human prostate cancer. Mol Carcinog.

[19] Saito Y, Kanai Y, Nakagawa T, Sakamoto M, Saito H, Ishii H, Hirohashi S (2003). Increased protein expression of DNA meth-yltransferase (DNMT) 1 is significantly correlated with the malignant potential and poor prognosis of human hepatocellular carcinomas. Int J Cancer.

[20] Girault I, Tozlu S, Lidereau R, Bièche I (2003). Expression analysis of DNA methyltransferases 1, 3A and 3B in sporadic breast carcinomas. Clin Cancer Res.

[21] Etoh T, Kanai Y, Ushijima S, Nakagawa T, Nakanishi Y, Sasako M, Kitano S, Hirohashi S (2004). Increased DNA methyltransferase 1 (DNMT1) protein expression correlates significantly with poorer tumor differentiation and frequent DNA hypermethylation of multiple CpG islands in gastric cancers. Am J Pathol.

[22] Lin RK, Hsu HS, Chang JW, Chen CY, Chen JT, Wang YC (2007). Alteration of DNA methyltransferases contributes to 5′CpG methylation and poor prognosis in lung cancer. Lung Cancer.

[23] Lujambio A, Lowe SW (2012). The microcosmos of cancer. Nature.

[24] Taddei ML, Giannoni E, Comito G, Chiarugi P (2013). Microenvironment and tumor cell plasticity: An easy way out. Cancer Lett.

[25] Gaudet F, Hodgson JG, Eden A, Jackson-Grusby L, Dausman J, Gray JW, Leonhardt H, Jaenisch R (2003). Induction of tumors in mice by genomic hypomethylation. Science.

[26] Narimatsu T, Tamori A, Koh N, Kubo S, Hirohashi K, Yano Y, Arakawa T, Otani S, Nishiguchi S (2004). p16 promoter hyper-methylation in human hepatocellular carcinoma with or without hepatitis virus infection. Intervirology.

[27] Zhang Z, Chen Y, Tang J, Xie X (2014). Frequent loss expression of dad2 and promotor hypermethylation in human cancers: A meta-analysis and systematic review. Pak J Med Sci.

[28] Yu G, Yao W, Gumireddy K (2014). Pseudogene PTENP1 functions as a competing endogenous RNA to suppress clear-cell renal cell carcinoma progression. Mol Cancer Ther.

[29] Christoforou N, Chellappan M, Adler AF, Kirkton RD, Wu T, Addis RC, Bursac N, Leong KW (2013). Transcription factors MYOCD, SRF, Mesp1 and SMARCD3 enhance the cardio-inducing effect of GATA4, TBX5, and MEF2C during direct cellular reprogramming. PLoS One.

[30] Zhu C, Shao P, Bao M (2014). miR-154 inhibits prostate cancer cell proliferation by targeting CCND2. Urol Oncol.

[31] Ogunwobi OO, Puszyk W, Dong HJ, Liu C (2013). Epigenetic upregulation of HGF and c-Met drives metastasis in hepatocellular carcinoma. PLoS One.

[32] Kivelä R, Bry M, Robciuc MR, Räsänen M, Taavitsainen M, Silvola JM, Saraste A, Hulmi JJ, Anisimov A, Mäyränpää MI (2014). VEGF-B-induced vascular growth leads to metabolic reprogramming and ischemia resistance in the heart. EMBO Mol Med.

[33] Koppenol WH, Bounds PL, Dang CV (2011). Otto Warburg's contributions to current concepts of cancer metabolism. Nat Rev Cancer.

[34] Suárez-Causado A, Caballero-Díaz D, Bertrán E (2015). HGF/c-Met signaling promotes liver progenitor cell migration and invasion by an epithelial-mesenchymal transition-independent, phosphatidyl inositol-3 kinase-dependent pathway in an in vitro model. Biochim Biophys Acta.

[35] Lordick F (2014). Targeting the HGF/MET pathway in gastric cancer. Lancet Oncol.

[36] Tischoff I, Tannapfe A (2008). DNA methylation in hepatocellular carcinoma. World J Gastroenterol.

[37] Egger G, Liang G, Aparicio A, Jones PA (2004). Epigenetics in human disease and prospects for epigenetic therapy. Nature.

[38] Massagué J, Blain SW, Lo RS (2000). TGFbeta signaling in growth control, cancer and heritable disorders. Cell.

[39] Dong C, Wu Y, Yao J, Wang Y, Yu Y, Rychahou PG, Evers BM, Zhou BP (2012). G9a interacts with Snail and is critical for Snail-mediated E-cadherin repression in human breast cancer. J Clin Invest.

[40] Yang MH, Hsu DS, Wang HW, Wang HJ, Lan HY, Yang WH, Huang CH, Kao SY, Tzeng CH, Tai SK (2010). Bmi1 is essential in Twist1-induced epithelial-mesenchymal transition. Nat Cell Biol.

[41] Tong ZT, Cai MY, Wang XG, Kong LL, Mai SJ, Liu YH, Zhang HB, Liao YJ, Zheng F, Zhu W (2012). EZH2 supports nasopharyngeal carcinoma cell aggressiveness by forming a co-repressor complex with HDAC1/HDAC2 and Snail to inhibit E-cadherin. Oncogene.

[42] Jones PA, Baylin SB (2002). The fundamental role of epigenetic events in cancer. Nat Rev Genet.

[43] Wang D, Chang PS, Wang Z, Sutherland L, Richardson JA, Small E, Krieg PA, Olson EN (2001). Activation of cardiac gene expression by myocardin, a transcriptional cofactor for serum response factor. Cell.

[44] Failli V, Rogard M, Mattei MG, Vernier P, Rétaux S (2000). Lhx9 and Lhx9alpha LIM-homeodomain factors: Genomic structure, expression patterns, chromosomal localization and phylogenetic analysis. Genomics.

[45] Vladimirova V, Mikeska T, Waha A, Soerensen N, Xu J, Reynolds PC, Pietsch T (2009). Aberrant methylation and reduced expression of LHX9 in malignant gliomas of childhood. Neoplasia.

[46] Lai CP, Bechberger JF, Naus CC (2009). Pannexin2 as a novel growth regulator in C6 glioma cells. Oncogene.

